# Autoimmune encephalitis associated with anti-LGI1 antibody: a potential cause of neuropsychiatric systemic lupus erythematosus

**DOI:** 10.1136/lupus-2024-001429

**Published:** 2025-02-18

**Authors:** Sixian Chen, Haitao Ren, Siyuan Fan, Shangzhu Zhang, Mengtao Li, Hongzhi Guan

**Affiliations:** 1 Neurology, Peking Union Medical College Hospital, Beijing, China; 2 Department of Rheumatology and Clinical Immunology, Peking Union Medical College Hospital, Beijing, China; 3 Department of Rheumatology, Peking Union Medical College Hospital, Beijing, China; 4 Peking Union Medical College Hospital, Beijing, China

**Keywords:** Lupus Erythematosus, Systemic, Antibodies, Autoimmune Diseases

## Abstract

**Objective:**

To investigate the clinical features and treatment outcomes of anti-leucine-rich glioma-inactivated 1 (anti-LGI1) encephalitis in patients with SLE.

**Methods:**

Between October 2014 and April 2024, serum or cerebrospinal fluid samples were collected from 332 patients with SLE suspected of autoimmune encephalitis. Cell-based assays were used to detect autoimmune antibodies, including anti-LGI1 antibodies. Four patients tested positive for anti-LGI1 antibodies, and their clinical, radiological and treatment data were analysed.

**Results:**

All four patients exhibited signs of limbic encephalitis, including short-term memory deficits, seizures and psychiatric disturbances. Two cases also presented with faciobrachial dystonic seizures. MRI findings revealed hyperintense basal ganglia lesions in two patients. Treatment with corticosteroids, intravenous immunoglobulin and mycophenolate mofetil led to significant improvement in three patients, with no relapses during a follow-up period ranging from 33 to 60 months. One patient succumbed to pneumonia despite initial improvement of neurological function.

**Conclusion:**

Screening for anti-LGI1 antibodies in patients with neuropsychiatric systemic lupus erythematosus (NPSLE) is crucial when limbic encephalitis presents, as it enables timely and effective treatment, potentially improving patients’ outcomes. Additional basic and clinical research is required to clarify the pathogenic role of these antibodies in NPSLE.

**WHAT IS ALREADY KNOWN ON THIS TOPIC**

- Autoimmune encephalitis is increasingly recognised in autoimmune diseases, with anti-neural antibodies such as anti-N-methyl-D-aspartate receptor (anti-NMDAR) and anti-leucine-rich glioma-inactivated 1 (anti-LGI1) playing a pathogenic role.
- Neuropsychiatric systemic lupus erythematosus (NPSLE) is a complex and heterogeneous condition, and its classification and diagnosis remain challenging.

**WHAT THIS STUDY ADDS**

- This study is the first to report cases of anti-LGI1 encephalitis in patients with SLE, demonstrating its clinical features and treatment outcomes.
- It highlights the importance of screening for anti-LGI1 antibodies in patients with NPSLE with limbic encephalitis symptoms.

**HOW THIS STUDY MIGHT AFFECT RESEARCH, PRACTICE OR POLICY**

- This study suggests that revising NPSLE diagnostic criteria is urgent.
- It also underscores the need for autoimmune encephalitis-associated antibody screening to improve diagnosis and treatment, encouraging further research into the underlying mechanisms.

##  Introduction

SLE is a chronic, systemic autoimmune disease characterised by the involvement of multiple organs and systems. Neuropsychiatric systemic lupus erythematosus (NPSLE), a significant clinical phenotypes of SLE, has been classified into 19 distinct phenotypes, 12 of which are associated with the central nervous system according to the American College of Rheumatology.^1^ However, with the identification of pathogenic anti-neural antibodies as diagnostic biomarkers for autoimmune neurological disease, an increasing number of cases are being recognised as comorbidities or supplements, including neuromyelitis optica spectrum disorders (NMOSD) caused by anti-aquaporine 4 antibodies.^2^

In recent years, significant progress has been made in the study of anti-neuronal antibodies, such as anti-N-methyl-D-aspartate receptor (anti-NMDAR) and anti-leucine-rich glioma-inactivated 1 (anti-LGI1) antibodies, which have been confirmed as pathogenic antibodies for autoimmune encephalitis.^3^ To date, there have been no reported cases of SLE with anti-LGI-1 encephalitis. This article presents four relevant cases.

## Method

### Patients and samples

From October 2014 to April 2024, serum or cerebrospinal fluid (CSF) samples from 332 patients with SLE with clinically suspected autoimmune encephalitis were collected and analysed by the Neuroimmunology and Encephalitis Laboratory of Peking Union Medical College Hospital (PUMCH). Cell-based assays were employed to detect autoimmune encephalitis antibodies and paraneoplastic antibodies, including anti-NMDAR/LGI1/GABAb-R/CASPR2/GAD65/AMPAR antibodies and anti-Hu/Yo/Ri/CV2/Tr/Ma2/Amphiphysin antibodies (EUROIMMUN, Lübeck, Germany). Among these patients, four were identified with positive anti-LGI1 antibodies in either serum or CSF (table 1).

**Table 1:** Clinical characteristics of four patients of SLE with anti-LGI1 encephalitis

No.		1	2	3	4
Gender/age		F/30	M/67	F/69	M/65
Seizures		FBDS; GTCS	+, no FBDS	FBDS	GTCS
Psychiatric symptoms		+	+	–	–
Cognitive dysfunction		+	+	+	+
Short-term memory deficit		+	+	+	+
dLoC		+	+	+	+
Hyponatremia		+	+	–	+
Systemic autoantibody	ANA	1:320	1:100	1:320	1:160
	Anti-dsDNA	–	–	+	–
	Anti-SSA	+	–	+	–
	Anti-SSB	–	–	+	–
	Anti-rRNP	–	–	–	–
	Anti-RNP	–	–	–	–
	aPLs	–	–	–	+
CSF leucocyte counts (10×10^6^/L)		0	NA	4	2
SOB		+	NA	–	NA
Anti-LGI1	CSF	1:10	NA	1:32	1:100
	Serum	–	1:100	1:320	1:32
Neuroimaging	Hyperintensity on the T1-weighted imaging in the basal ganglia	+	–	+	–
	Limbic lesions of MRI	+	–	–	+
	Other imagings	–	–	–	^18^F-FDG PET/CT identified asymmetrically increased metabolism in the swelling caudate nucleus and putamen.
EEG	Epileptiform activity	+	–	–	NA
	Slow waves	–	–	–	NA
Treatment	IVIg	+	–	+	+
	High-dose steroids	+	–; only 60 mg/day	–; only 60 mg/day	+
	Immunosuppressive drugs	CTX; MMF	MMF	MMF	MMF
Prognosis		Complete restoration	Seizure-free; mild short-term memory impairment	Significant improvements in memory and mental status, with no seizures	Died because of severe pneumonia and respiratory failure
Follow-up months		60	33	44	12

18PET/CT, 18CT; aPLs, antiphospholipid antibodies; CSF, cerebrospinal fluid; CTX, cyclophosphamide; dLoC, decreased level of consciousness; EEG, electroencephalogram; FBDS, faciobrachial dystonic seizures; GTCS, generalised tonic-clonic seizures; IVIg, IV immunoglobulin; MMF, mycophenolate mofetilNA, not available; SOB, specific oligoclonal bands

## Result

### Case 1

In 2012, a woman in her early 30s diagnosed with lupus nephritis was effectively managed on low-dose prednisolone (5 mg/day) without any notable symptoms. Her medical history was unremarkable. In January 2019, she began to experience diarrhoea. A week later, she was admitted with short-term memory deficits at another hospital, progressing to disorganised speech, hallucinations and transient, jerky right-sided facial and arm involuntary movements, followed by altered consciousness. Initial investigations revealed hyponatraemia (serum sodium: 97 mmol/L) and a normal leucocyte count in CSF.

Treatment with IV immunoglobulin (IVIg) at 0.4 g/kg/day, high-dose methylprednisolone (1 g/day) and antiepileptics (carbamazepine and sodium valproate) yielded gradual cognitive and psychiatric improvement. However, somnolence persisted. Subsequent CSF analysis conducted at our hospital identified anti-LGI1 antibodies (titre 1:3.2). In March, she experienced recurrent faciobrachial dystonic seizures (FBDS) occurring at a frequency of one episode per minute, alongside generalised tonic-clonic seizures, with refractory hyponatraemia. Laboratory results showed ANA S1:320, positive anti-SSA antibodies, low C3/C4, negative antiphospholipid antibodies. On April 22, lumbar puncture showed no leucocytes and a protein concentration of 0.23 g/L, positive cerebrospinal fluid specific oligoclonal bands (SOB), negative paraneoplastic antibody and positive anti-LGI1 antibody in CSF samples at titre of 1:10 while negative in serum samples.

Brain MRI showed a hyperintense signal on T1-weighted imaging in the left basal ganglia and T2-weighted hyperintense lesions in the right insular lobe. Electroencephalogram (EEG) revealed epileptiform activity in the left frontotemporal region. Continued treatment with corticosteroids, IVIg and cyclophosphamide led to substantial clinical recovery. The patient was discharged with a reduced frequency of FBDS and normalised MRI findings. Over 60 months, she has been regularly taking mycophenolate mofetil and achieved complete functional restoration (Systemic Lupus Erythematosus Disease Activity Index 2000 (SLEDAI-2K)=0, modified Rankin Scale (mRS)=0).

### Case 2

A man in his late 60s underwent a skin biopsy in 2016 due to significant hair loss, which suggested discoid lupus. The presence of ANA at a titre of 1:100 and reduced levels of C3 and C4 led to the consideration of SLE. In July 2021, the patient experienced limb convulsions and episodes of clouded consciousness lasting several minutes. He also developed intermittent symptoms characterised by vivid imaginary images, followed by palpitations, which resolved after more than 10 seconds. Initially, these episodes occurred more than 10 times per day, eventually increasing to several dozen, but improved with antiepileptic therapy. Additional symptoms included emotional blunting, short-term memory deficits and diminished computational ability. Serum anti-LGI1 antibodies were detected at a titre of 1:100. Lumbar puncture was not performed. Brain MRI and EEG showed no abnormalities. The patient was treated with oral prednisone (60 mg/day) and long-term mycophenolate mofetil. After 33 months of follow-up, the patient remained seizure-free after the discontinuation of steroids and antiepileptic drugs, although mild short-term memory impairment persisted (SLEDAI-2K=0, mRS=0).

### Case 3

**Figure 1:**
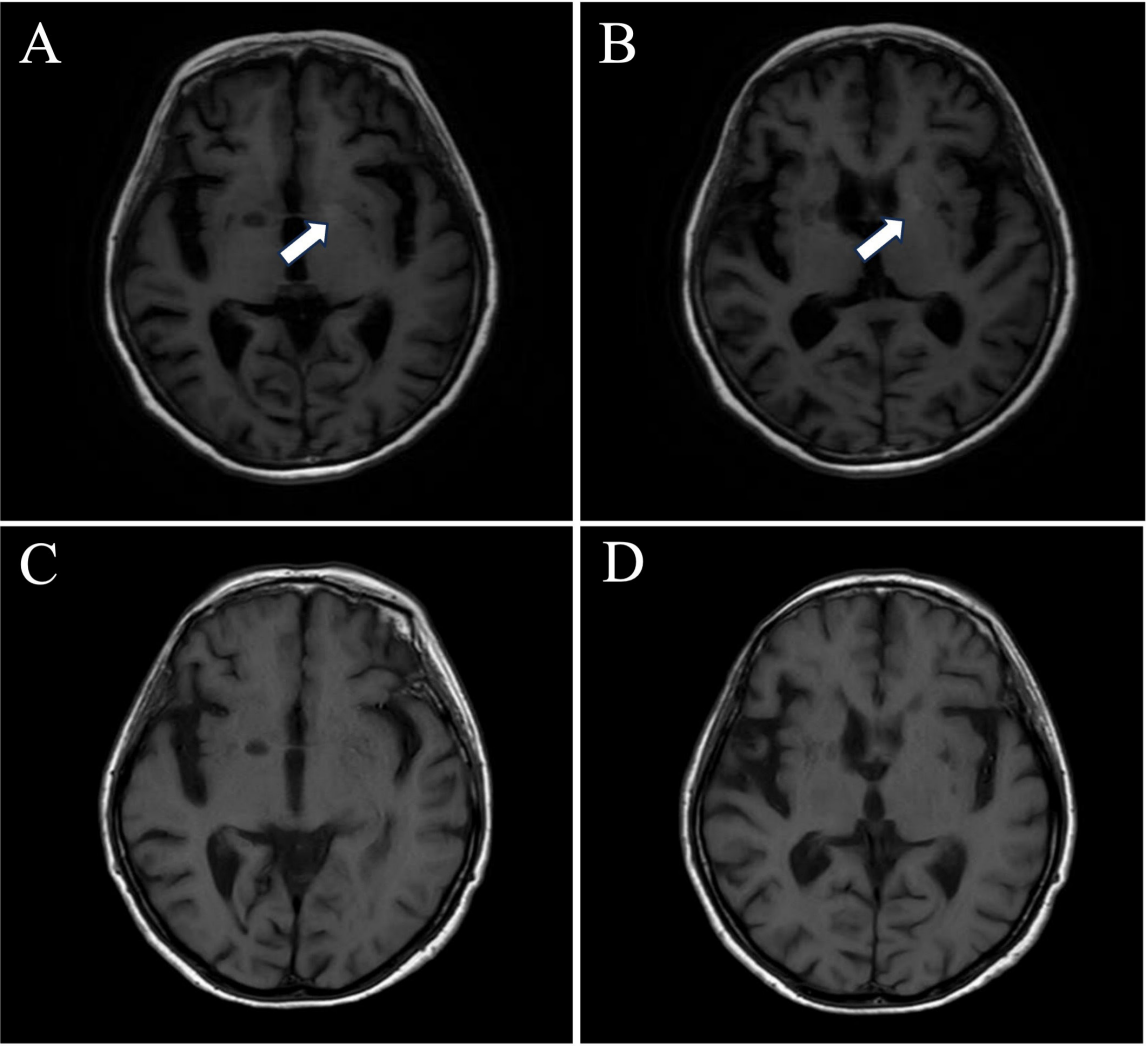
Brain MRI of case 3 showed T1-weighted hyperintensities in the left basal ganglia (A, B, marked by white arrows). The hyperintense signal on T1-weighted imaging in the left basal ganglia has resolved (C) after 44 months of follow-up.

A woman in her late 60s, with a 17-year history of SLE, exhibited delayed responsiveness and short-term memory deficits in July 2020. On 8 August 2020, she experienced twitching of the right hand and the right side of the mouth, lasting 20 seconds to 1 minute, over 20 times daily. Laboratory tests revealed ANA at 1:320, positive anti-dsDNA, anti-SSA and anti-SSB antibodies, negative antiphospholipid antibodies, and serum anti-LGI1 antibody 1:320. Lumbar puncture showed 4×10^6^/L leucocytes, negative SOB and anti-LGI1 antibody 1:32. Brain MRI showed T1-weighted hyperintensities in the left basal ganglia (figure 1). Interictal EEG was unremarkable. Treatment included IVIg at 0.4 g/kg/day for 5 days, prednisone at 60 mg/day, antiepileptic therapy with levetiracetam and sodium valproate, and long-term oral mycophenolate mofetil. After 44 months of follow-up, the patient showed significant improvements in memory and mental status, with no seizures (SLEDAI-2K=0, mRS=0). Her serum anti-LGI-1 antibody levels turned into negative and the T1-weighted hyperintensities in the left basal ganglia has resolved.

### Case 4

**Figure 2:**
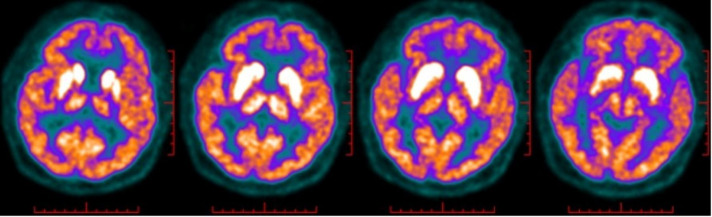
^18^F-fluorodeoxyglucose positron emission tomographic/computed tomographic imaging of case 4 identified asymmetrically hypermetabolism in the swelling caudate nucleus and putamen.

A man in his mid-60s, previously diagnosed with SLE in 2012, began experiencing a decreased level of consciousness and frequent generalised tonic-clonic seizures in mid-July 2015. Laboratory tests revealed hyponatraemia (Na 118 mmol/L), and lumbar puncture results indicated 2×10^6^/L leucocytes, protein levels at 0.71 g/L. Anti-LGI1 antibodies were detected in both the CSF (1:100) and serum (1:32), while paraneoplastic antibodies were negative. Brain MRI showed significant atrophy in the right temporal lobe, and ^18^F-fluorodeoxyglucose positron emission tomographic/computed tomographic imaging identified asymmetrically increased metabolism in the swollen caudate nucleus and putamen (figure 2). On admission, the patient presented with drowsiness and scored 9 on the Montreal Cognitive Assessment, with no other neurological abnormalities. Additional tests indicated ANA at 1:160, positive anti-β2-GP1 antibodies, leucopenia, thrombocytopenia, a positive Coomb’s test, but no evidence of haematological diseases on bone marrow biopsy. Treatment included IVIg, high-dose methylprednisolone followed by prednisone, sodium supplementation and antiepileptic drugs, leading to improved sodium levels and no seizures. Maintenance therapy with mycophenolate mofetil was administered. Unfortunately, the patient succumbed to severe pneumonia and respiratory failure 1 year later.

## Discussion

To date, there have been reports of SLE with NMOSD, anti-NMDAR encephalitis^4^ and anti-AMPAR encephalitis,^5^ but no reports of SLE with anti-LGI-1 encephalitis. We report four cases of anti-LGI-1 encephalitis in patients with SLE.

The diagnosis and management of lupus encephalitis/encephalopathy remain particularly challenging. Anti-neural antibodies have been identified as key diagnostic biomarkers for autoimmune diseases of the central nervous system, such as the pathogenic roles of anti-NMDAR and anti-LGI1 antibodies in autoimmune encephalitis, and anti-AQP4 antibodies in NMOSD. The current diagnostic criteria for NPSLE do not exclude patients who test positive for anti-neural antibodies. Therefore, these patients can still meet the diagnostic criteria for NPSLE, posing a challenge for disease diagnosis.

Most patients with anti-LGI1 antibodies exhibit symptoms of limbic encephalitis, marked by a subacute onset of memory and behavioural disturbances, often with seizures.^6^ Specifically, FBDS are indicative of anti-LGI1 encephalitis. All four patients in this study exhibited classic symptoms of limbic encephalitis; two of the patients also presented with FBDS.

Both patients in Case 1 and Case 3 exhibited FBDS-associated hyperintense signals on T1-weighted imaging in the basal ganglia, which resolved as their conditions improved. This suggests a correlation between this imaging marker and disease activity, consistent with previous reports.^7^ However, the mechanism remains unclear. Compared with patients with anti-NMDAR encephalitis, those with anti-LGI1 encephalitis do not often show elevated leucocyte counts in CSF,^8^ making the screening for autoimmune encephalitis antibody panel crucial for diagnosis. Most patients have a good prognosis with timely and adequate immunotherapy.^9^

Autoimmune diseases can exhibit overlapping clinical phenotypes, which may suggest the presence of common pathogenic antibodies. Anti-LGI1 antibodies, as one of the confirmed pathogenic antibodies for autoimmune encephalitis, are produced by peripheral B cells.^10^ These antibodies cross the blood-brain barrier^11^ and interfere with the interaction between LGI1 and presynaptic ADAM23 and postsynaptic ADAM22. This disruption reduces the levels of presynaptic Kv1.1 and postsynaptic AMPAR, increasing glutamatergic synaptic transmission, and leading to short-term memory deficits and seizures.^12^ Since the pathogenic antibodies of neuropsychiatric lupus are not yet clear, it remains controversial whether these neuroimmune diseases are comorbidities or if such anti-neural antibodies could be considered pathogenic antibodies for NPSLE or whether they could be classified as a neurological phenotype of NPSLE.^13^,^15^ Further research is needed to confirm this.

Currently, the classification of neuropsychiatric lupus is mostly based on the 1999 American College of Rheumatology (ACR) nomenclature and case definitions,^1^ which, in addition to the diagnostic challenges described earlier, have other limitations.

First, the 1999 ACR case definitions are not aimed at the neurological aetiology of SLE but rather lists symptoms according to central and peripheral nervous systems, resulting in definitional overlap. The aetiology behind a particular symptom in patients with SLE is diverse. For example, cerebrovascular disease or aseptic meningitis (broadly speaking, when grey matter damage is also involved) can both present with seizures. Besides, headaches can also be a clinical manifestation of aseptic meningitis.

What’s more, there is a considerable amount of definitional ambiguity. For example, seizures are listed as focal syndromes of NPSLE. Given that epilepsy can be classified into focal onset and generalised onset, distinguishing whether the seizures are focal or diffuse in lupus is urgent. Therefore, updating the classification of neuropsychiatric lupus is necessary. If autoimmune encephalitis could be classified as a new phenotype of neuropsychiatric lupus, our suggestion of diagnostic criteria can be referred to in box 1.

Box 1 **Suggestion of diagnostic criteria for autoimmune encephalitis as a phenotype of neuropsychiatric systemic lupus erythematosus**

Diagnosis can be made when all five* of the following criteria have been met:

1. Diagnosis of systemic lupus erythematosus: Confirmed according to SLICC-2012 or EULAR/ACR-2019 Classification Criteria.
2. Neurological clinical presentation: Acute or subacute onset (rapid progression within less than 3 months), with at least one of the following symptoms:
  - Limbic system involvement: Short-term memory deficits, seizures, or psychiatric and behavioural abnormalities.
  - Encephalitis syndrome: Clinical manifestations of diffuse or multifocal brain damage.
  - Basal ganglia and/or diencephalon/hypothalamus involvement: Clinical signs indicating affected regions.
3. Auxiliary findings: At least one of the following:
  - CSF pleocytosis (white blood cell count >5×10^6^/L)
  - Lymphocytic inflammation in CSF cytology
  - Positive specific oligoclonal bands
  - Brain T2 or FLAIR abnormal signals in the medial temporal lobes (unilateral or bilateral), and/or multiple cortical and/or basal ganglia regions†
  - Abnormal EEG showing focal epilepsy or epileptiform discharges (in the temporal lobe or extratemporal areas) or slow-wave rhythms with diffuse or multifocal distribution, or extreme delta brush
4. Positive anti-neural antibodies: Detected in serum or cerebrospinal fluid through indirect immunofluorescence assay.
5. Reasonable exclusion of alternative causes (infection, metabolism, tumour, heredity, etc).

*If anti-neural antibodies are absent, the diagnosis can only be classified as possible neuropsychiatric systemic lupus erythematosus autoimmune encephalitis.†An increase uptake in ¹⁸F-fluorodeoxyglucose positron emission tomographic/computed tomographic imaging in these regions can be used to fulfil this criterion.ACR, American College of Rheumatology; CSF, cerebrospinal fluid; EEG, electroencephalogram; EULAR, the European League Against Rheumatism; FLAIR, fluid-attenuated inversion recovery; SLICC, Systemic Lupus International Collaborating Clinics.

The patients described in this article primarily presented with limbic encephalitis, and the principles for treatment differ significantly from other aetiologies that might cause similar symptoms. A more refined classification of neuropsychiatric lupus will provide clearer insights into the underlying aetiologies of the disease.

In summary, screening for autoimmune encephalitis-related antibodies in patients with NPSLE is necessary for timely and appropriate treatment, potentially improving prognosis. Future basic and clinical research is warranted to elucidate the pathogenic role of autoimmune encephalitis-related antibodies in patients with NPSLE.

## Data Availability

Data are available upon reasonable request.
